# Ferroptosis: A Key Driver in Atherosclerosis Progression and Arterial Disease

**DOI:** 10.31083/j.rcm2512441

**Published:** 2024-12-17

**Authors:** Amr Elkammash, Abrar Zaki, Omar Tawfik, Sherif Gouda

**Affiliations:** ^1^Department of Cardiology, Bristol Heart Institute, BS2 8HW Bristol, UK; ^2^Department of General Medicine, Eastbourne District General Hospital, BN21 2UD East Sussex, UK; ^3^Department of Cardiology, Royal Gwent Hospital, NP20 2UB Newport, UK

**Keywords:** ferroptosis, iron overload, chelation, reactive oxygen species, Fenton reaction, atherosclerosis

## Abstract

Atherosclerosis (AS) is a growing global health epidemic and is the leading cause of cardiovascular health problems, including ischemic stroke, coronary artery disease, and peripheral vascular disease. Despite extensive research on the underlying mechanisms of AS, iron remains an under-investigated mediator in the atherosclerotic process. Iron’s involvement in AS is primarily linked to the iron-induced programmed cell death process known as ferroptosis. Ferroptosis is initiated in endothelial cells when iron overload triggers the Fenton reaction, resulting in the production of reactive oxygen species (ROS) and lipid peroxides. This oxidative stress damages cellular components, ultimately leading to cell death. The review examines the role of iron overload and ferroptosis in the progression and instability of atherosclerotic plaques. Additionally, we explore the potential therapeutic roles of iron chelators and ROS scavengers in mitigating the adverse effects of ferroptosis. The findings indicate that ferroptosis contributes significantly to the progression and instability of atherosclerotic plaques by promoting oxidative damage and cellular dysfunction. Iron chelators and ROS scavengers have shown promise in reducing ferroptosis-induced damage in endothelial cells. These therapeutic agents can potentially stabilize atherosclerotic plaques and prevent the progression of AS. Ferroptosis is a critical yet under-explored pathway in the development and progression of atherosclerosis. Targeting iron-induced oxidative stress through iron chelation and ROS scavenging presents a promising therapeutic strategy for mitigating the adverse effects of ferroptosis on atherosclerotic plaque stability. Further research is needed to validate these therapeutic approaches and better understand the molecular mechanisms underlying ferroptosis in atherosclerosis.

## 1. Introduction

Iron overload conditions, such as hemochromatosis and transfusion-related iron overload, present a unique risk for the induction of ferroptosis [1]. Ferroptosis is an iron-dependent form of regulated cell death characterized by the accumulation of lipid peroxides to lethal levels. Recent studies have illuminated its pivotal role in various pathological conditions, including cancer, neurodegeneration, and ischemia-reperfusion injury. The exploration of ferroptosis in cardiovascular diseases has gained momentum, with a growing body of evidence suggesting its significant involvement in the progression of atherosclerosis (AS). AS, a leading cause of cardiovascular morbidity and mortality worldwide, is driven by the interplay of lipid accumulation, chronic inflammation, and cell death within the arterial wall. Understanding the mechanisms by which ferroptosis contributes to atherosclerotic lesion formation and progression is crucial for identifying novel therapeutic targets [2, 3].

Recent advancements in the field have highlighted the dual role of ferroptosis in AS. On one hand, ferroptosis of endothelial and smooth muscle cells can exacerbate plaque vulnerability by compromising the structural integrity of the arterial wall. On the other hand, the targeted induction of ferroptosis in foam cells and macrophages within plaques has been proposed as a strategy to mitigate atherosclerotic burden by clearing pathogenic lipid-laden cells [4]. These findings demonstrate the complexity of ferroptosis regulation in AS, suggesting that therapeutic modulation of this cell death pathway could offer a double-edged sword in treating cardiovascular diseases.

Emerging research has also identified key molecular players and signaling pathways that regulate ferroptosis in AS. For instance, the role of glutathione peroxidase 4 (GPX4), a critical inhibitor of lipid peroxidation, has been extensively studied, revealing its protective effects against ferroptosis-induced cell death in atherosclerotic lesions. Moreover, the interplay between iron metabolism and lipid oxidation presents a novel area of exploration for potential therapeutic interventions. As we delve deeper into the molecular underpinnings of ferroptosis in AS, it becomes imperative to develop targeted strategies that can modulate this complex cell death pathway to prevent or treat atherosclerotic cardiovascular diseases [5].

## 2. The Role of Ferroptosis in the Progression of Atherosclerosis

**Fig. 1.**
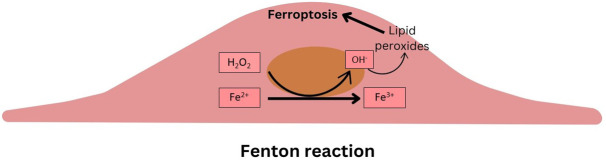
**Schematic summarizing the Fenton reaction within the intimal cells**.

Iron, an essential element for various physiological processes, can catalyze the Fenton reaction when in excess, leading to oxidative stress and cytotoxicity (Fig. 1). Regulation of iron import, storage, and export is therefore crucial for maintaining cellular redox balance and preventing ferroptosis. Studies have shown that disturbances in iron homeostasis, such as through the action of ferritinophagy, can significantly influence ferroptosis and, by extension, AS [3, 6].

At the core of ferroptosis’s impact on atherosclerosis is the accumulation of lipid reactive oxygen species (ROS), which is induced by an elevated intracellular iron concentration, depletion of antioxidants like glutathione, or the presence of atherosclerosis inducers such as oxidized low-density lipoprotein (ox-LDL) [7].

There are four primary categories of fatty acids: saturated, monounsaturated, polyunsaturated (PUFAs), and trans fats. Among these, PUFAs, which contain multiple carbon-carbon double bonds, are closely linked to ferroptosis due to their high susceptibility to oxidation [8]. Their abundance of double bonds makes them particularly prone to oxidative damage by ROS. This oxidative stress primarily targets membrane-bound PUFAs, leading to the generation of free radicals that exacerbate cellular damage [9].

Ether phospholipids (ePLs), a distinct class of phospholipids characterized by an ether bond at the sn-1 position of the glycerol backbone, are also implicated in ferroptosis due to their susceptibility to peroxidation. Unlike ester phospholipids, ePLs are more vulnerable to ROS, which can lead to the accumulation of lipid peroxides and promote ferroptosis [10]. Mechanisms that reduce oxidized ePLs, remove lipid peroxides from membranes, or suppress ether lipid peroxidation have been shown to protect against ferroptosis. The levels of ePLs influence the selective susceptibility of certain cells or tissues to ferroptosis in their membranes [11].

Additionally, the signaling pathways involving NRF2-Keap1 (NRF2, nuclear factor erythroid 2-related factor 2, is a transcription factor that controls the expression of a wide range of genes involved in antioxidant defense, detoxification, and cellular protection. It activates the transcription of genes that help to protect cells from oxidative damage, inflammation, and various stressors. Keap1, Kelch-like ECH-associated protein 1, is a negative regulator of NRF2) [12] and p53 (a tumour suppressor gene that induces apoptosis and promotes DNA repair) [13] play crucial roles in modulating ferroptosis in the context of AS. The NRF2-Keap1 pathway, for example, helps decrease ferroptosis associated with AS by maintaining cellular iron homeostasis and increasing the production of antioxidants [14]. The p53 pathway suppresses the expression of *SLC7A11*, a crucial part of the cystine/glutamate antiporter that facilitates cystine transport and prevents ROS-induced ferroptosis [15].

## 3. Iron Overload and Ferroptosis

In the context of cardiovascular diseases, iron overload has been linked to an accelerated progression of AS, as the oxidative stress generated by ferroptosis can exacerbate endothelial dysfunction and plaque instability. Vinchi *et al*. [16] have shown that patients with conditions leading to systemic iron overload exhibit a higher prevalence of atherosclerotic lesions, underscoring the detrimental role of iron in vascular health.

Iron accumulation within atherosclerotic plaques has been observed to potentiate the ferroptotic pathway, further contributing to plaque progression and vulnerability [1]. The role of iron in facilitating ferroptosis within atherosclerotic lesions has been corroborated by animal models, where iron chelation was found to mitigate plaque development and stabilize plaque morphology, highlighting the potential therapeutic value of targeting iron homeostasis in atherosclerosis prevention [17].

## 4. Treatment Targets to Delay the Progression of Atherosclerosis via the Management of the Cellular Iron Load

Therapeutic strategies to mitigate iron overload and inhibit ferroptosis have shown promise in decelerating atherosclerotic disease progression. The use of iron chelators, such as Deferiprone, has demonstrated a reduction in lipid peroxidation and a subsequent decrease in ferroptotic cell death within atherosclerotic plaques [18]. Additionally, manipulating key ferroptosis regulators, such as GPX4 and system xc-, offers a targeted approach to curtail lipid peroxidation and protect against iron-induced cellular damage [19]. These findings confirm the critical interplay between iron metabolism and ferroptosis in the context of AS and suggest that modulating iron levels could serve as a viable strategy to prevent or treat atherosclerotic cardiovascular diseases.

El Hajj *et al*. [20] demonstrated a successful reduction of intracellular lipid peroxidation and cellular ferroptosis using the iron chelator Deferiprone in a smooth muscle-based ferroptosis model. However, Deferoxamine (another iron chelator) did not have the same protective effect [20]. On the contrary, Bai *et al*. [21] showed the effectiveness of Deferoxamine in stopping cellular lipid peroxidation and ferroptosis. Herbal iron chelators ( such as Flavinoids) were effective in attenuating the progression of AS, activating the NRF2 signaling pathway, AMPK (AMPK, adenosine monophosphate-activated protein kinase, is a key energy-sensing enzyme that regulates cellular energy homeostasis) signaling pathway, and KAT5/GPX4 signaling pathway (KAT5, lysine acetyltransferase 5, is a gene that regulates DNA repair and chromatin remodeling), thereby reducing iron overload and lipid peroxidation in cardiovascular diseases [22]. Impairment of lipid peroxides production using the Glutathione pathway has demonstrated effectiveness in providing cardiovascular protection in *ex-vivo* models [23].

## 5. Conclusions

Ferroptosis, a form of regulated cell death driven by lipid peroxidation, plays a significant role in the progression of atherosclerotic arterial disease. Iron overload amplifies this process by catalyzing the formation of lipid peroxides, thereby accelerating vascular damage and plaque development. Targeting ferroptosis through the use of iron chelators and agents that boost the glutathione pathway could offer protective effects against atherosclerosis progression. Nevertheless, further randomized human trials are needed to confirm the potential benefits and safety of these therapeutic approaches.
